# Dissolved organic monomer partitioning among bacterial groups in two oligotrophic lakes

**DOI:** 10.1111/1758-2229.12240

**Published:** 2015-01-23

**Authors:** María Teresa Pérez, Carina Rofner, Ruben Sommaruga

**Affiliations:** ^1^ Lake and Glacier Research GroupInstitute of EcologyUniversity of InnsbruckTechnikerstrasse 256020InnsbruckAustria

## Abstract

Understanding how resource partitioning works among taxa is crucial in explaining coexistence and competition within a community. Here, we assessed resource partitioning among freshwater bacterial groups from two oligotrophic lakes using four types of organic substrates as compound models. Substrate uptake patterns were examined by microautoradiography combined with catalysed reporter deposition fluorescent *in situ* hybridization. Four large taxonomic groups were found in the lakes, but *A* *ctinobacteria* (AcI lineage) and *B* *etaproteobacteria* (R‐BTcluster) dominated the bacterial assemblage. Monomers containing nitrogen and/or phosphorus were preferred over the ones containing only carbon. All groups were able to incorporate amino acids, adenosine triphosphate and glucose. However, acetate was only taken up by ∼ 10–12% of bacteria, and its uptake was not detected in *C* *ytophaga*‐*F* *lavobacteria*. Apart from acetate, the contribution of a particular bacterial group to the uptake of a substrate was proportional to its relative abundance. In both lakes, we detected substrate partitioning between AcI *Actinobacteria*, which was overrepresented in glucose and acetate utilization, and R‐BT *B* *etaproteobacteria*, which dominated amino acid uptake. Our results strongly point to physiological niche separation of those bacterial groups in alpine lakes.

## Introduction

Heterotrophic bacteria largely rely on the labile fraction of the dissolved organic matter (DOM) that is characterized by short turnover times of hours to days (Kirchman *et al*., 1991; Cherrier *et al*., 1996). Although components of this labile DOM fraction are usually found at nanomolar concentrations, they are important in supporting a large fraction of bacterial production and growth in marine and freshwater systems (Kirchman, 2003).

In both types of aquatic ecosystems, the bacterial assemblage is usually dominated by just a few large taxonomical groups. In freshwaters, for instance, *Betaproteobacteria* and *Actinobacteria* have been found to be the prevailing groups (Methé *et al*., 1998; Glöckner *et al*., 2000), although they show contrasting temporal dynamics with Actinobacterial peaks appearing at times of reduced Betaproteobacterial abundance (Glöckner *et al*., 2000; Burkert *et al*., 2003; Pérez and Sommaruga, 2011).

Within the class *Actinobacteria*, the AcI lineage is the most abundant one in freshwaters, and often comprises > 90% of this group (Warnecke *et al*., 2005; Allgaier and Grossart, 2006). In turn, the contribution of the R‐BT cluster to *Betaproteobacteria* is more variable, but it can reach up to 80% of *Betaproteobacteria* (Pérez *et al*., 2010; Šimek *et al*., 2010). More interesting is the fact that in lakes, this group can drive the response of the entire bacterial community in terms of biomass production (Šimek *et al*., 2005; 2006; Pérez and Sommaruga, 2006).

*Betaproteobacteria* and *Actinobacteria* have apparently different life strategies. Whereas *AcI Actinobacteria* have been qualified as defence specialists (Salcher, 2014) due to their small cell size that protects them from grazing (Jezbera *et al*., 2006; Šimek *et al*., 2006), *Betaproteobacteria* and particularly the fast‐growing R‐BT subgroup were associated to an opportunistic lifestyle with resource specialization and high vulnerability to predation (Salcher, 2014). However, recently Thingstad and colleagues (2014) pointed out that the key to numerical success might just be an intermediate strategy, in which the right balance between defensive and competitive capabilities is reached.

In the last decade, several studies have examined the uptake of diverse substrates by either large taxonomic groups or individual bacterial populations, in marine as well as in freshwaters. Whereas in marine studies a variety of substrates has been tested (Cottrell and Kirchman, 2000; Malmstrom *et al*., 2005; Alonso‐Sáez and Gasol, 2007), in freshwaters most studies have focused on the use of amino acids either as leucine or as mixtures (Hornák *et al*., 2006; Pérez and Sommaruga, 2006; Salcher *et al*., 2010). However, the study of alternative substrates by Buck and colleagues (2009) indicated substrate partitioning between two Betaproteobacterial clades (R‐BT and PnecC) in a humic lake. Similarly, we found that in alpine lakes, the R‐BT cluster of *Betaproteobacteria* was underrepresented in thymidine uptake, whereas *Actinobacteria* dominated the uptake of this substrate (Pérez *et al*., 2010). However, whether these bacterial groups preferentially take up other low molecular weight compounds is not known.

Here, we present results from experiments addressed to determine the use of four model compounds by the principal bacterial groups of two clear alpine lakes: Lake Gossenköllesee (GKS) and Lake Schwarzsee ob Sölden (SOS). Substrate uptake patterns were examined by microautoradiography (MAR) combined with catalysed reporter deposition fluorescent *in situ* hybridization (CARD‐FISH). We considered the following substrate families – amino acids, carboxylic acids, neutral sugars and phosphoesters – because they are commonly found in a wide range of aquatic systems and can support large fractions of bacterial production (Kirchman, 2003). Although phosphorus limitation is a common feature in freshwaters, so far no study has investigated the use of phosphorous compounds by individual freshwater bacterial groups.

We particularly addressed whether substrate partitioning between the R‐BT subgroup of *Betaproteobacteria* and the Actinobacterial lineage AcI could help explain the observed codominance of the bacterial assemblage by these lineages in alpine oligotrophic lakes during the ice‐free season (Warnecke *et al*., 2005; Pérez and Sommaruga, 2011). Additionally, we investigated the use of these substrates by other, less abundant but recurrent, taxonomic groups, namely *Cytophaga*‐*Flavobacteria* and *Alphaproteobacteria*.

## Results and discussion

### Sampling conditions and bacterial community composition

We selected two clear water oligotrophic alpine lakes as experimental sites. Lake GKS was sampled in September 2006 at 1 m depth (GKS 1 m) and 8.5 m depth (GKS 8.5 m), and SOS was sampled at 1 m depth in September 2006 (SOS 06) and in August 2007 (SOS 07). Dissolved organic carbon (DOC) concentrations were low (< 40 μM) in both lakes (Table 1) but are typical for alpine lakes in this region (Laurion *et al*., 2000). The lakes differed greatly in their nitrogen content. Total dissolved nitrogen concentrations were two to three times lower in SOS than in GKS, whereas total dissolved phosphorus (TDP) concentrations were similar in both lakes (Table 1), with the exception of the epilimnion of GKS where TDP concentration was only 0.02 μM.

**Table 1:** Basic physicochemical characterization of the samples from GKS (collected in September 2006 at 1 m and 8.5 m depth respectively) and SOS (collected at 1 m depth in September 2006 and August 2007 respectively)

Sample	Temp	pH	DOC	TDN	TDP	N : P
	(°C)		(μM)	(μM)	(μM)	Molar ratio
GKS 1 m	11.2	7.16	39.9	16.5	0.02	825
GKS 8.5 m	8.7	6.97	33.5	18.3	0.06	305
SOS 06	7.5	6.08	36.6	6.93	0.04	173
SOS 07	9.2	6.18	34.2	6.29	0.05	126

Temp, water temperature; DOC, dissolved organic carbon; TDP, total dissolved phosphorus; TDN, total dissolved nitrogen. TDP analyses were done as described by Psenner (1989).

Details on the bacterial assemblage composition at the time of sampling can be found in Pérez and colleagues (2010). As expected, *Betaproteobacteria* and *Actinobacteria* were equally abundant in GKS (each *c*. 20% of DAPI counts). In SOS, *Actinobacteria* dominated in the first year (∼ 38% DAPI counts), but on the subsequent year *Betaproteobacteria* was the most abundant group (∼ 42% of DAPI counts). This suggests that the high relative abundances of *Actinobacteria* (∼ 70% DAPI counts) found previously in this lake (Warnecke *et al*., 2005) are not a permanent feature of its bacterial assemblage. Noteworthy were the proportions of *Betaproteobacteria* that we detected with probe R‐BT065 (Šimek *et al*., 2005) in both lakes (60% to 80% of *Betaproteobacteria* in GKS and SOS respectively). Those proportions are among the highest reported to date (Šimek *et al*., 2010). For instance, the R‐BT subgroup represented between 24% and 33% of total DAPI counts in SOS, so far one of the highest relative abundances ever reported for this cluster (Šimek *et al*., 2010). This is interesting because the R‐BT cluster, now part of the *Limnohabitans* genus, is among the fastest growing freshwater bacteria (Šimek *et al*., 2006) and easily overgrows other bacterial groups under favourable conditions (Šimek *et al*., 2005; Pérez and Sommaruga, 2006). Additionally, R‐BT bacteria have been found to be the main drivers of bacterial activity and production in GKS (Pérez and Sommaruga, 2011), and thus they probably play a key role in DOM cycling in alpine lakes.

The *Actinobacteria* class was dominated by the AcI lineage, which represented on average 93% of the Actinobacterial cells. This is in good agreement with the results of Warnecke and colleagues (2005) for several mountain lakes in the central Alps (Tirol), and of Buck and colleagues (2009) and Allgaier and Grossart (2006) for lakes from the Mecklenburg‐Brandenburg Lake District, Germany. Although *Cytophaga*‐*Flavobacteria* and *Alphaproteobacteria* were less abundant, they also were represented in the bacterial community in both lakes.

### Substrate uptake patterns at the community level

The bacterial assemblage of both lakes was remarkably active in the uptake of monomers, with the exception of acetate (Table 2). Particularly, the relative abundance of active cells involved in amino acid uptake in Lake SOS is the highest ever reported so far. This suggests that the bacterial community of alpine lakes is very well adapted to their habitat despite the harsh environmental conditions they experience (Sommaruga, 2001). Our results on acetate contrast with those of Buck and colleagues (2009), who found that cells active for acetate uptake comprised 2.5–46.5% of bacteria. However, the acetate concentration used by these authors (10 nM) was five to ten times higher than ours.

**Table 2:** Relative abundance of active cells (expressed as percentage of total DAPI counts) found in samples from GKS and SOS with the substrates tested (AA, ATP, GLU and ACET)

Sample	% Active LEU	% Active AA	% Active ATP	% Active GLU	% Active ACET
	(% DAPI)	(% DAPI)	(% DAPI)	(% DAPI)	(% DAPI)
GKS 1 m	65.9 (4.71)	53.9 (5.63)	65.3 (2.60)	50.0 (1.19)	10.0 (0.66)
GKS 8.5 m	49.1 (3.37)	67.5 (4.81)	67.6 (4.40)	48.2 (1.58)	10.6 (0.63)
SOS 06	71.2 (3.36)	82.5 (2.48)	74.6 (3.07)	52.9 (1.55)	14.7 (0.27)
SOS 07	67.2 (4.49)	81.9 (2.37)	72.5 (1.63)	50.7 (0.92)	12.3 (0.92)

Activity was assessed by means of microautoradiography after incubating the samples for 5 h with one of the following ^3^H‐radiolabelled substrates (AA, amino acids; ATP, adenosine triphosphate; GLU, glucose; ACET, acetate). All substrates were offered at a final concentration of 1–2 nM. Leucine (LEU) was offered at saturating concentration (20 nM). Data on LEU from Pérez and colleagues (2010) are included here for comparison. Values in between brackets correspond to the SD of triplicate samples.

Overall, we observed that compounds composed of carbon and phosphorus or carbon and nitrogen were preferentially taken up by the bacterial assemblage as a whole, as compared with those composed solely of carbon (Table 2). Studies considering bulk uptake rates by the whole bacterial community have found that dissolved free amino acids can support a high proportion of bacterial production (up to 90–100%) in both marine (Nagata, 2008) and freshwaters ecosystems (Kirchman, 2003). By contrast, glucose uptake in freshwaters has been found to support a smaller fraction (1–21%) of bacterial production (Kirchman, 2003), and our results are in agreement with this finding. Studies on the use of adenosine triphosphate (ATP) as substrate are scarce, and they all refer to marine ecosystems. For instance, in the phosphorus‐limited north‐west Mediterranean Sea, ATP is preferentially taken up by bacteria compared with amino acids and glucose (Alonso‐Sáez and Gasol, 2007). Similarly, in GKS (Table 2), higher or similar proportions of cells took up ATP than the amino acid mixture, but the opposite held true for SOS. A plausible explanation about the amino acid mixture preference in SOS is difficult to find. A tentative explanation could be provided by the stoichiometry of the major nutrients (Table 1). Thus, the N : P ratio in GKS is extremely high averaging 550, whereas in SOS it is ∼ 150, indicating that phosphorus is not as scarce in SOS as compared with nitrogen as it seems to be the case in GKS, particularly in the epilimnion of the lake.

### Substrate uptake patterns at the group level

To determine absolute substrate preferences at the group level can be a tricky task as those preferences may vary from system to system (Salcher *et al*., 2010; 2013), and depend among other factors on the substrate concentrations offered (Alonso and Pernthaler, 2006) and the specific activity of the substrate (Fuhrman and Azam, 1982). Thus, here we just focused on substrate partitioning between the very abundant *Betaproteobacteria* (R‐BT cluster) and AcI *Actinobacteria*. To do so, the four substrates considered in this study were offered at trace concentrations (1–2 nM), which are likely to be near their *in situ* concentration in alpine lakes with very low DOC content (35–40 μM). Indeed, in our ongoing work in GKS, we have found that the average ATP concentration in the lake is < 3 nM (C. Rofner, R. Sommaruga and M. T. Pérez, unpubl. data). Similarly, glucose and DFAA concentrations in other oligotrophic aquatic systems have been found to be in the low nanomolar range (Skoog *et al*., 1999; Sempéré *et al*., 2008; Zubkov *et al*., 2008). Although the acetate concentrations measured by Buck and colleagues (2009) in a humic lake were high (∼ 10 μM), we still decided to offer acetate at a trace concentration to enable comparison with the other monomers used.

**Figure 1:**
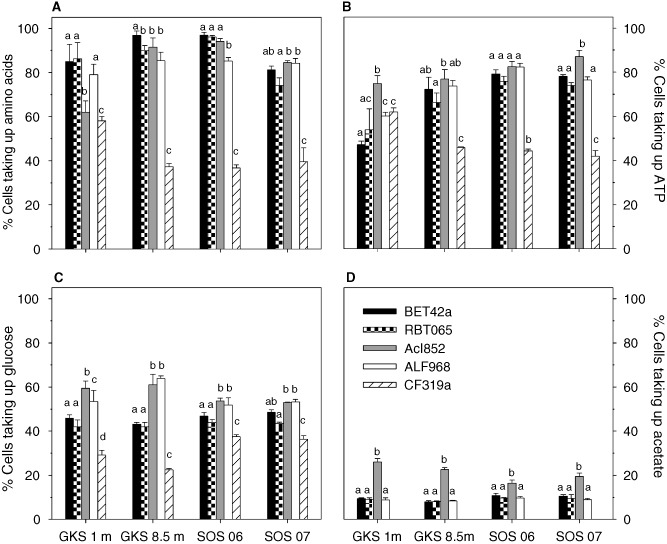
Relative abundance of cells active (% hybridized cells) within the most abundant bacterial groups: *B* *etaproteobacteria* (BET42a) and its R‐BT cluster (RBT065), AcI *A* *ctinobacteria* (AcI852), *A* *lphaproteobacteria* (ALF968) and *C* *ytophaga*‐*F* *lavobacteria* (CF319a) for amino acid (A), ATP (B), glucose (C) and acetate (D) uptake. All substrates were offered at 1–2 nM concentration. Details on the MAR‐CARD‐FISH procedure can be found in Appendix S1. The letter code above the bars summarizes the results of the *post hoc* all pairwise multiple comparison test (significance level of 0.05) used to detect significant differences in substrate utilization among bacterial groups. Values are mean of three replicates ± 1 SD.

Amino acid uptake was observed in all bacterial groups examined, albeit the proportions of active cells varied among groups (Fig. 1A). In agreement with previous reports (Cottrell and Kirchman, 2000; Hornák *et al*., 2006; Pérez and Sommaruga, 2006), *Cytophaga‐Flavobacteria* was found to harbour the lowest proportions of cells active in amino acid uptake. By contrast, *Betaproteobacteria* showed always the highest proportion of active cells (up to 97% of hybridized cells). *Betaproteobacteria* and its R‐BT cluster are usually very active components of the bacterial community in amino acid uptake regardless of the concentration tested (Salcher *et al*., 2008; Pérez *et al*., 2010; Pérez and Sommaruga, 2011).

ATP uptake was also detected in all examined groups (Fig. 1B). AcI *Actinobacteria* showed always high proportions of active cells (up to 87% of hybridized cells) closely followed by *Betaproteobacteria* and *Alphaproteobacteria*. Although in most cases the relative abundance of active *Cytophaga‐Flavobacteria* was significantly lower as compared with other groups, ATP was still the substrate that allowed the detection of the highest proportions of cells active within this group (up to ∼ 62% of hybridized cells). This is in good agreement with the results of Longnecker and colleagues (2010), who also found higher proportions of active *Cytophaga‐Flavobacteria* using a phosphorus containing substrate as marker compared with other substrates, such as leucine and thymidine.

Two bacterial groups showed significantly higher proportions of active cells in glucose uptake, namely AcI *Actinobacteria* and *Alphaproteobacteria* (Fig. 1C). By contrast, less than 50% of hybridized *Betaproteobacteria* and R‐BT cells were found positive with this marker. Our results resemble those obtained by Hornák and colleagues (2010) and Salcher and colleagues (2013), who also found high proportions of active *Actinobacteria* for glucose uptake. AcI *Actinobacteria* (Fig. 1D) was also the group that showed the highest proportion of active cells for acetate. By contrast, acetate uptake was not detected in *Cytophaga‐Flavobacteria* (Fig. 1D).

**Figure 2:**
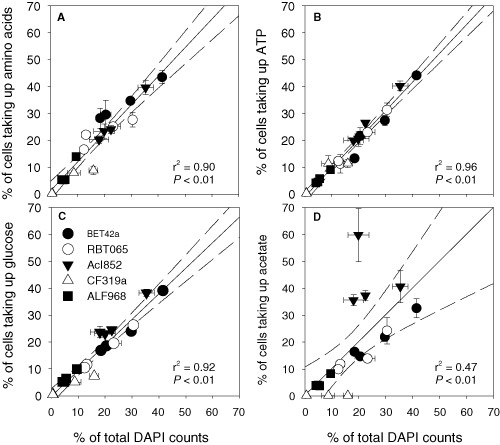
Relative contribution of *B* *etaproteobacteria* (BET42a), R‐BT cluster (RBT065), AcI *A* *ctinobacteria* (AcI852), *C* *ytophaga*‐*F* *lavobacteria* (CF319a) and *A* *lphaproteobacteria* (ALF968) to amino acid (A), ATP (B), glucose (C) and acetate (D) uptake plotted against their relative abundance (% total DAPI counts). For details on the MAR‐CARD‐FISH procedure, check Appendix S1. Solid lines are linear regression lines and dashed lines indicate 95% confidence intervals. The corresponding regression coefficients and their statistical probabilities are presented on the plots. Values are mean of three replicates ± 1 SD.

An essential aspect to understand the role of distinct bacterial groups in the uptake of monomers *in situ* is to determine how they contribute to substrate uptake as compared with their relative abundance. Here, we found that bacterial community composition explained up to 96% of the variability in ATP uptake (Fig. 2B and Table S1), and ∼ 90% of the variability in glucose and amino acid uptake (Fig. 2A and C and Table S1). However, for acetate (Fig. 2D and Table S1), only 47% of the variability in its uptake was related to the relative abundance of bacterial groups, with Ac I *Actinobacteria* being overrepresented in the uptake of this substrate. These results are somehow different from those of Buck and colleagues (2009) working in a humic lake, where they found *Betaproteobacteria* to dominate acetate uptake. Given the very different nature of the lake they studied and taking into account that *Polynucleobacter necessarius* (PnecC; Hahn *et al*., 2005), and not the R‐BT cluster of *Betaproteobacteria*, was the most active group in acetate uptake in that system, allows for reconciliation of our apparent contradictory results.

### Which bacterial groups ‘dominate’ the uptake of different monomers?

**Figure 3:**
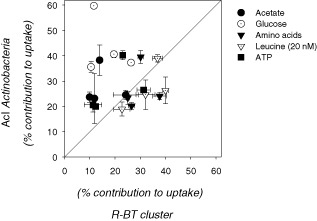
Fraction of active cells (acetate, glucose, amino acids, leucine and ATP) within the dominant bacterial groups: AcI *A* *ctinobacteria* versus the R‐BT cluster of *B* *etaproteobacteria*, normalized to the fraction of total active cells. The black line (1:1) defines both groups as being equally important in the uptake of the considered substrate. Distance from the line indicates an overrepresentation of one of the bacterial groups in the uptake of a particular monomer. Data from leucine were taken from Pérez and colleagues (2010) and plotted here for comparison.

As the contribution of bacterial groups to the uptake of a particular substrate was best explained by their relative abundance, one could just expect no dominant bacterial group to be overrepresented in the uptake of any of the substrates. However, a closer look to Fig. 3 indicates that most of the cells active in acetate and glucose uptake belong to the AcI lineage of *Actinobacteria*. In contrast, the R‐BT cluster of *Betaproteobacteria* (Fig. 3) dominated the uptake of the amino acid mixture and leucine (data from Pérez *et al*., 2010, plotted here for comparison).

Our results on glucose and amino acid partitioning between *Actinobacteria* and *Betaproteobacteria* are in good agreement with those of Hornák and colleagues (2010). These authors found that in the Řimov Reservoir, Czech Republic, *Betaproteobacteria* is the prevalent group in leucine uptake, whereas *Actinobacteria* play an important role in the uptake of glucose. Interestingly, in our study, all bacterial groups contributed to ATP uptake proportionally to their abundance, suggesting that the acquisition of dissolved organic phosphorus is an important strategy to live in phosphorus‐limited lakes.

Whether bacteria are generalists able to utilize multiple carbon compounds and its possible consequences for the ecosystem functioning has been a matter of great debate in recent literature (Mou *et al*., 2008; Poretsky *et al*., 2010; Gómez‐Consarnau *et al*., 2012). A generalist strategy has been postulated to be advantageous in oligotrophic systems to exploit the apparently homogenous and nutrient‐poor environment (Egli, 2010). However, most semi‐quantitative studies combining MAR with CARD‐FISH have found differences in substrate uptake patterns among bacterial groups in both marine and freshwater oligotrophic ecosystems (Malmstrom *et al*., 2005; Alonso‐Sáez and Gasol, 2007; Pérez *et al*., 2010). For instance, in marine waters, SAR 11 has been shown to dominate amino acid uptake (Malmstrom *et al*., 2004; 2005). Similarly, in freshwaters, the R‐BT subgroup of *Betaproteobacteria* has been found to dominate the uptake of leucine but was underrepresented in the uptake of thymidine (Pérez *et al*., 2010).

Our present results, although based on only four substrates, strongly point out to substrate partitioning between the abundant bacterial lineages found in oligotrophic alpine lakes. The dominance of *Betaproteobacteria*, and to a large extent of its R‐BT subgroup in amino acid uptake, seems to be a recurrent and thus a strong feature of this group in alpine lakes and freshwaters, in general. By contrast, *Actinobacteria* seem to rely more on alternative substrates, such as sugars and/or carboxylic acids (Hornák *et al*., 2010). The narrower substrate preferences by AcI *Actinobacteria* have been also pointed out by recent genomic and experimental studies (Garcia *et al*., 2013; Salcher *et al*., 2013). On the contrary, *Limnohabitans* members (including the R‐BT cluster) seem to have a higher metabolic flexibility (Zeng *et al*., 2012; Salcher *et al*., 2013). The differences in substrate uptake we observed here can be interpreted as a physiological niche separation and considered as an important factor explaining the coexistence of abundant bacterial clusters like the AcI *Actinobacteria* and R‐BT cluster in freshwaters. However, the right balance between competitive and defensive strategies might be the secret to numerical success in a community (Thingstad *et al*., 2014).

## Supporting information


**Table S1.** Relative contribution of the main bacterial groups to the uptake of amino acids, ATP, acetate and glucose expressed as % of total active cells. GKS 1 m and GKS 8.5 m represent samples collected in GKS at 1 m and 8.5 m depth. SOS 06 and SOS 07 are samples collected in SOS at 1 m depth in 2006 and 2007. BET (*Betaproteobacteria*), AcI (AcI lineage of *Actinobacteria*), ALF (*Alphabroteobacteria*) and CF (*Cytophaga*‐*Flavobacteria*). Values are means of three replicates. Numbers in brackets correspond to the standard deviation. n. d. not detected.

**Appendix S1.** Supplementary experimental procedures.
