# Supplementary material for: Dissolved organic monomer partitioning among bacterial groups in two oligotrophic lakes
Source: Environ Microbiol Rep. 2015 Jan 23;7(2):265–72. doi: 10.1111/1758-2229.12240 (PMC4452937; doi:10.1111/1758-2229.12240)
Supplement: Supplementary file 1 — Table S1. Relative contribution of the main bacterial groups to the uptake of amino acids, ATP, acetate and glucose expressed as % of total active cells. GKS 1 m and GKS 8.5 m represent samples collected in GKS at 1 m and 8.5 m depth. SOS 06 and SOS 07 are samples collected in SOS at 1 m depth in 2006 and 2007. BET (Betaproteobacteria), AcI (AcI lineage of Actinobacteria), ALF (Alphabroteobacteria) and CF (Cytophaga‐Flavobacteria). Values are means of three replicates. Numbers in brackets correspond to the standard deviation. n. d. not detected. [file EMI4-7-265-s001.docx]

Table S1. Relative contribution of the main bacterial groups to the uptake of amino acids, ATP, acetate and glucose expressed as % of total active cells. GKS 1 m and GKS 8.5 m represent samples collected in GKS at 1 m and 8.5 depth. SOS 06 and SOS 07 are samples collected in SOS at 1 m depth in 2006 and 2007. BET (*Betaproteobacteria*), AcI (AcI lineage of *Actinobacteria*), ALF (*Alphabroteobacteria*) and CF (*Cytophaga*-*Flavobacteria*). Values are means of three replicates. Numbers in brackets correspond to the standard deviation. n. d. not detected.

| Sample | Amino Acids | | | | ATP | | | | Acetate | | | | Glucose | | | |
| --- | --- | --- | --- | --- | --- | --- | --- | --- | --- | --- | --- | --- | --- | --- | --- | --- |
|  | BET | AcI | ALF | CF | BET | AcI | ALF | CF | BET | AcI | ALF | CF | BET | AcI | ALF | CF |
|  |  |  |  |  |  |  |  |  |  |  |  |  |  |  |  |  |
| GKS 1 m | 28.3  (3.73) | 23.4  (2.62) | 14.0  (1.47) | 7.85  (0.77) | 13.3  (0.77) | 20.6  (2.82) | 9.14  (0.94) | 11.3  (2.92) | 16.4  (1.41) | 59.8 (9.87) | 8.23 (1.25) | n. d. | 16.9  (1.27) | 23.2 (0.99) | 9.94  (0.71) | 5.15  (0.47) |
| GKS 8.5 m | 29.5  (5.41) | 20.2  (2.62) | 5.42  (0.76) | 8.84  (1.52) | 21.7  (3.07) | 20.0  (0.80) | 4.18  (0.33) | 11.4  (1.47) | 14.7  (1.39) | 35.6  (1.99) | 3.88 (0.19) | n. d. | 18.6  (2.89) | 23.7  (2.11) | 5.23  (0.56) | 7.34  (0.49) |
| SOS 06 | 3.47  (0.82) | 39.6  (2.51) | 5.40  (0.51) | 0.24  (0.01) | 27.4(  1.86) | 40.2  (1.80) | 5.75  (0.69) | 0.40  (0.03) | 21.9  (1.69) | 40.6 (5.99) | 3.75  (0.15) | n. d. | 24.0  (4.13) | 38.3  (1.34) | 6.39  (0.68) | 0.51  (0.04) |
| SOS 07 | 43.5  (2.45) | 24.1  (1.47) | 5.32  (0.17) | 0.21  (0.03) | 44.1  (0.28) | 26.5  (0.57) | 4.52  (0.40) | 0.30  (0.01) | 32.6  (3.55) | 37.3  (1.78) | 3,99  (0.32) | n. d. | 39.1  (2.28) | 24.5  (1.17) | 5.25  (0.60) | 0.39  (0.05) |
|  |  |  |  |  |  |  |  |  |  |  |  |  |  |  |  |  |
