# Supplementary material for: Dissolved organic monomer partitioning among bacterial groups in two oligotrophic lakes
Source: Environ Microbiol Rep. 2015 Jan 23;7(2):265–72. doi: 10.1111/1758-2229.12240 (PMC4452937; doi:10.1111/1758-2229.12240)
Supplement: Supplementary file 2 — Appendix S1. Supplementary experimental procedures. [file EMI4-7-265-s002.docx]

**Dissolved organic monomer partitioning among bacterial groups in two oligotrophic lakes**

María Teresa Pérez, Carina Rofner, and Ruben Sommaruga

**Supplementary Experimental Procedures**

**Sampling sites and sample collection**

Water samples were collected during the ice-free season from two alpine lakes located in the Austrian Alps. Lake Gossenköllesee (GKS) is a small lake situated at 2417 m above sea level. The maximum depth in its central area, where the samples were collected, is 9.9 m. Lake Schwarzsee ob Sölden (SOS) is located at 2799 m (a. s. l.) and has a maximum depth of 18 m. Triplicate water samples from GKS were collected at 1 m depth and at 8.5 m in September 2006, whereas SOS was sampled at 1 m depth twice, in September 2006 and in August 2007. Other characteristics of these lakes can be found in Laurion *et al.* ([2000](#_ENREF_22)). At every sampling date, temperature, pH, dissolved organic carbon (DOC) and nutrient concentrations were measured. DOC and total dissolved nitrogen (TDN) samples were filtered through two pre-combusted (4h 450ºC) GF/F filters. The filtrate was acidified to pH 2 and analyzed with a Shimadzu TOC-Vc series equipped with a total nitrogen module.

**Incubations for micro-autoradiography**

Triplicate (10-15 ml) water samples and a formaldehyde-killed blank were incubated in the dark with one of the following ^3^H-labeled compounds (Amersham): acetate (6 Ci mmol^-1^), ATP (50 Ci mmol^-1^), glucose (33 Ci mmol^-1^) and an amino acid mixture (45 Ci mmol^-1^). The amino acid mixture was commercially available from Amersham (UK) and contained 15 different amino acids. Among them five amino acids (leu, ile, ala, glu and ser) represented *ca.* 56% of the total mixture. The substrates were added at trace concentrations (1-2 nM) and incubated for 5 hours at *in situ* temperature. Incubations ended with the addition of formaldehyde (2% final concentration) to the samples. Samples were kept at 4 °C overnight and then filtered onto 0.22 µm white polycarbonate filters (Millipore GTTP), rinsed with 0.22 µm filtered Milli-Q water, air dried and then stored at -20°C until analysis.

**CARD-FISH and micro-autoradiography analysis**

CARD-FISH was done according to Pernthaler *et al.* (2002) using the permeabilization protocol of Sekar *et al.* (2003), modified for freshwater bacteria. We used the following horseradish peroxidase-labeled oligonucleotide probes : probe EUB I-III ([Daims *et al.*, 1999](#_ENREF_8)) targeting most *Bacteria*, BET42a ([Manz *et al.*, 1992](#_ENREF_24)) for *Betaroteobacteria*, R-BT065 ([Šimek *et al.*, 2001](#_ENREF_43)) for the R-BT subgroup of *Betaproteobacteri*a, HCG69a ([Roller *et al.*, 1994](#_ENREF_35)) for *Actinobacteria,* AcI852 ([Warnecke *et al.*, 2005](#_ENREF_47)) for the AcI clade of *Actinobacteria,* and CF319a ([Manz *et al.*, 1996](#_ENREF_23)) detecting most of *Cytophaga*-*Flavobacteria* of *Bacteroidetes*. All hybridizations were run at 35°C overnight, followed by 30 min amplification.

Following CARD-FISH procedure, cells were transferred to coverslips and subjected to microautoradiography (Cottrell and Kirchman, 2000). The optimum exposure time was assessed by monitoring the detection of positive cells for a given substrate until a maximum was reached. Slides were exposed for six (amino acid mixture), seven (ATP and glucose) or 10 days (acetate). After exposure, slides were developed according to the manufacturer instructions (Kodak) and mounted with an anti-fading solution (1x Citifluor, 2x Vectashield and 1x PBS) containing DAPI (final concentration 1 µg ml^-1^). Slides were stored frozen until the microscopy analysis.

Cells were counted in at least 20 randomly selected microscopic fields. Routinely, at least 400 DAPI-stained cells were counted per sample or 1000 DAPI-stained cells, when the calculated relative abundance was < 1%. All in all ca. 300 individual preparations were analyzed this way.

**References**

Cottrell, M.T., and Kirchman, D. (2000) Natural assemblages of marine proteobacteria and members of the Cytophaga-Flavobacter cluster consuming low and high molecular weight dissolved organic matter. *Appl Environ Microbiol* **66**: 1692-1697.

Daims, H., Bruhl, R., Amann, R., Schleifer, K.H., and Wagner, M. (1999) The domain-specific probe EUB338 is insufficient for the detection of all *Bacteria*: development and evaluation of a more comprehensive probe set. *Syst. Appl. Microbiol.* **22**: 434-444.

Laurion, I., Ventura, M., Catalan, J., Psenner, R., and Sommaruga, R. (2000) Attenuation of ultraviolet radiation in mountain lakes: Factors controlling the among- and within-lake variability. *Limnol Oceanogr* **45**: 1274-1288.

Manz, W., Amann, R., Ludwig, W., Wagner, M., and Schleifer, K.H. (1992) Phylogenetic oligodeoxynucleotide probes for the major subclasses of Proteobacteria: problems and solutions. *Syst Appl Microbiol* **15**: 593-600.

Manz, W., Amann, R., Ludwig, W., Vancanneyt, M., and Schleifer, K.H. (1996) Application of a suite of 16S rRNA-specific oligonucleotide probes designed to investigate bacteria of the phylum Cytophaga-Flavobacter-Bacteroides in the natural environment. *Microbiology* **142**: 1097-1106.

Pernthaler, A., Pernthaler, J., and Amann, R. (2002) Fluorescence *in situ* hybridization and catalyzed reporter deposition for the identification of marine bacteria. *Appl Environ Microbiol* **68**: 3094-3101.

Roller, C., Wagner, M., Amann, R., Ludwig, W., and Schleifer, K.H. (1994) In situ probing of gram-positive bacteria with high DNA G+C content using 23S rRNA-targeted oligonucleotides. *Microbiology* **140**: 2849-2858.

Sekar, R., Pernthaler, A., Pernthaler, J., Warnecke, F., Posch, T., and Amann, R. (2003) An improved method for quantification of freshwater *Actinobacteria* by fluorescence *in situ* hybridization. *Appl Environ Microbiol* **69**: 2928-2935.

Šimek, K., Pernthaler, J., Weinbauer, M.G., Hornák, K., Dolan, J.R., Nedoma, J. et al. (2001) Changes in bacterial community composition and dynamics and viral mortality rates associated with enhanced flagellate grazing in a mesoeutrophic reservoir. *Appl Environ Microbiol* **67**: 2723-2733.

Warnecke, F., Sommaruga, R., Sekar, R., Hofer, J.S., and Pernthaler, J. (2005) Abundances, identity and growth state of *Actinobacteria* in mountain lakes of different UV transparency. *Appl Environ Microbiol* **71**: 5551-5559.
